# Concise Review: Comparison of Culture Membranes Used for Tissue Engineered Conjunctival Epithelial Equivalents

**DOI:** 10.3390/jfb6041064

**Published:** 2015-12-11

**Authors:** Jon Roger Eidet, Darlene A. Dartt, Tor Paaske Utheim

**Affiliations:** 1 Department of Ophthalmology, Oslo University Hospital, Oslo 0424, Norway; 2 Schepens Eye Research Institute, Massachusetts Eye and Ear/Harvard Medical School, Boston, MA 02114, USA; E-Mail: Darlene_Dartt@meei.harvard.edu; 3 Department of Medical Biochemistry, Oslo University Hospital, Oslo 0424, Norway; E-Mail: utheim2@gmail.com; 4 Department of Oral Biology, University of Oslo, Oslo 0316, Norway; 5 Department of Ophthalmology, Vestre Viken Hospital Trust, Drammen 3004, Norway

**Keywords:** conjunctiva, epithelium, biomaterials, membranes, culture, tissue engineering, goblet cells, ocular surface, transplantation

## Abstract

The conjunctival epithelium plays an important role in ensuring the optical clarity of the cornea by providing lubrication to maintain a smooth, refractive surface, by producing mucins critical for tear film stability and by protecting against mechanical stress and infectious agents. A large number of disorders can lead to scarring of the conjunctiva through chronic conjunctival inflammation. For controlling complications of conjunctival scarring, surgery can be considered. Surgical treatment of symblepharon includes removal of the scar tissue to reestablish the deep fornix. The surgical defect is then covered by the application of a tissue substitute. One obvious limiting factor when using autografts is the size of the defect to be covered, as the amount of healthy conjunctiva is scarce. These limitations have led scientists to develop tissue engineered conjunctival equivalents. A tissue engineered conjunctival epithelial equivalent needs to be easily manipulated surgically, not cause an inflammatory reaction and be biocompatible. This review summarizes the various substrates and membranes that have been used to culture conjunctival epithelial cells during the last three decades. Future avenues for developing tissue engineered conjunctiva are discussed.

## 1. Conjunctiva

**Figure 1:**
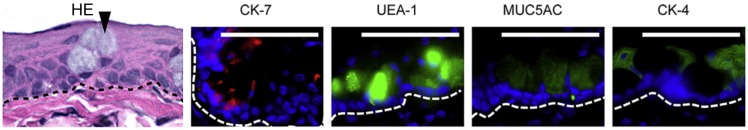
Photomicrographs show hematoxylin and eosin (HE) and immunofluorescently stained sections of rat conjunctiva. The black arrowhead in the HE photomicrograph indicates mucin granules of goblet cells. The black dotted line indicates the basal membrane, which overlies loose vascularized conjunctival forniceal connective tissue. Original magnification of the HE photomicrograph: ×630. Immunofluorescence photomicrographs of forniceal conjunctival sections show conjunctival epithelial cell markers, which include the goblet cell markers anti-cytokeratin 7 (Ck7), Ulex europaeus agglutinin 1 (UEA-1) lectin and anti-mucin 5AC (MUC5AC), as well as the marker for stratified squamous non-goblet cells anti-cytokeratin 4 (Ck4). Nuclei were stained with DAPI (blue). Ck7 is expressed in the goblet cell body, whereas UEA-1 and MUC5AC stain the goblet cell mucin-contents. Ck4 is only detected in squamous cells between goblet cell clusters. The basal membrane is indicated by the white dotted line. Scale bars: 100 μm. Adapted from Fostad *et al.* 2012 [7].

Conjunctival epithelium is non-keratinized and is at least two cell layers thick [1]. The number of cell layers depends on the degree of conjunctival stretching [2]. The conjunctival epithelium consists of two phenotypically distinct cell types, stratified squamous non-goblet cells (90%–95%) and goblet cells (5%–10%) (Figure 1), in addition to occasional lymphocytes [3] and melanocytes. The conjunctival epithelium plays an important role in ensuring the optical clarity of the cornea by providing lubrication to maintain a smooth, refractive surface, and by producing mucins critical for tear film stability [4]. The conjunctiva also protects the eye against mechanical stress and infectious agents. It, furthermore, contributes water and electrolytes to the tear fluid [5]. The squamous cells produce cell membrane-tethered mucins, while the goblet cells secrete the gel-forming mucins, both of which helps to maintain a protective tear film. The superficial surface of the squamous cells are covered by the membrane-tethered mucins mucin-1 (MUC1), mucin-4 (MUC4) and mucin-16 (MUC16) [6], which are essential for tear stability and make up the glycocalyx [6].

**Figure 2:**
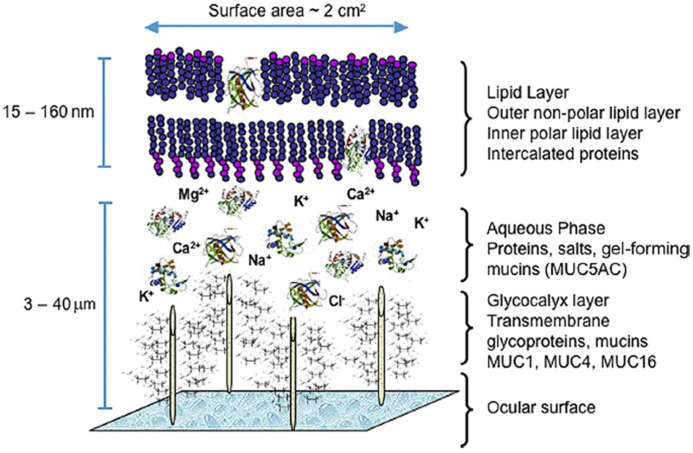
Model of the human tear film. Adapted from Nichols *et al.* 2001 [12].

The gel-forming mucin-5AC (MUC5AC) and mucin-2 (MUC2) are secreted by goblet cells into the aqueous layer of the tear film [8,9] (Figure 2). The squamous conjunctival cells also contribute to the hydration of the ocular surface through ion transport across the apical cell membrane with accompanying osmotic water transfer [5]. Goblet cells contain mucin-granules and have traditionally been identified through their secretory product using markers, including the ulex europaeus agglutinin-1 (UEA-1) lectin, anti-mucin-5AC (MUC5AC) and anti-AM3 antibodies, and periodic acid-Schiff (PAS) reagent that target the goblet cell gel-forming mucins [10]. In addition to cytokeratin 4 (Ck4) (Figure 1), squamous conjunctival epithelial cells can be identified by Ck13, a binding pair of Ck4 [11].

## 2. Conjunctival Stem Cells

Conjunctival stem cells continuously regenerate the conjunctiva by giving rise to both stratified squamous non-goblet and goblet cells [13], thereby maintaining a healthy tear film [14]. Disorders that damage these stem cells cause varying extent of keratinization, which disrupts the protective tear film and ultimately leads to limbal stem cell deficiency (LSCD) and visual impairment or blindness. The location of the conjunctival epithelial stem cells has been investigated in several studies on mouse [15,16,17], rat [18,19], rabbit [20,21] and human [22,23,24] tissue, yet no real consensus has been reached. The conjunctival stem cells have been suggested to reside in the limbus [18], bulbar conjunctiva [15,22,23], medial canthal [24], forniceal conjunctiva [16,17,20,22,24,25], palpebral conjunctiva [19] and mucocutaneous junction [18,21]. Although the conjunctival stem cells may not solely be located to one single region, their relative number generally appears to be highest in the fornix [26].

Stem cells are surrounded and influenced by a three-dimensional microenvironment known as a niche [27]. The niche comprises of numerous components, including stromal cells, soluble factors, extracellular matrix (ECM), mechanical/spatial cues and signaling molecules that dictates stem cell function [28]. The limbal stem cell niche has been reported to contain specific ECM proteins. Moreover, the specific composition of the ECM shows topographical variations throughout the ocular surface [29]. Thus, the specific composition of the ECM in the substrate may affect the preservation of conjunctival stem cells in culture.

## 3. Conjunctival Scarring Diseases

A large number of disorders can lead to scarring of the conjunctiva through chronic conjunctival inflammation. Scarring varies in severity and can be self-limited, such as in chemical/thermal burns and infectious diseases due to adeno- and herpes viruses, or progressive, as in cicatrizing conjunctivitis, which consists of several diseases including ocular cicatricial pemphigoid, Stevens-Johnson syndrome, atopic keratoconjunctivitis and Sjögren’s syndrome [30]. Cicatrizing conjunctivitis is rare, and in total these disorders have an incidence of 1.2 in 1 million in the United Kingdom [30]. Treatment depends on the disease etiology and severity, but can include various anti-inflammatory, immunomodulatory and immunosuppressive drugs [31].

Surgical treatment of symblepharon includes removal of the scar tissue to reestablish the deep fornix [32]. The surgical defect is then covered with a tissue substitute to prevent re-obliteration. These include mechanical [33], physical [34] or chemical [35] approaches and the grafting of conjunctival or mucous membranes [32]. Surgical techniques for restoration of a diseased conjunctiva have utilized different conjunctival substitutes, including conjunctival autografts [36]. An obvious limiting factor when using autografts is the size of the defect to be covered, as the amount of healthy conjunctiva is limited. These drawbacks have led scientists to develop tissue engineered conjunctival equivalents.

## 4. Tissue Engineered Conjunctival Equivalents

A tissue engineered conjunctival epithelial equivalent needs to be easily manipulated surgically, not cause an inflammatory reaction, be biocompatible and contain a mix of stratified squamous cells, goblet cells and undifferentiated cells. Unlike tissue engineered corneal equivalents, conjunctival equivalents do not need to be transparent, which increases the range of suitable culture membranes.

In addition to conjunctival epithelial cells (CEC) cultured on amniotic membrane (AM) [4], there is likely a wide range of cell types that can be used for developing a tissue engineered conjunctival equivalent. This assumption is based on multiple studies demonstrating successful restoration of the cornea with cultured non-limbal cells. Tissue engineered corneal equivalents share many of the same prerequisites as conjunctival equivalents, e.g., with regard to barrier function and tear film support. Besides limbal stem cells, corneal equivalents have been developed from oral mucosal epithelial cells [37,38], embryonic stem cells (ESC) [39], bone-marrow-derived mesenchymal stem cells (MSC) [40], immature dental pulp stem cells [41], hair follicle-derived stem cells [42] and umbilical cord lining stem cells [43]. For conjunctival reconstruction, epidermal keratinocytes have been cultured on AM and transplanted to restore the conjunctiva in rhesus monkeys [44]. Although the conjunctival stratified squamous cell markers MUC4 and Ck4 were present in the transplant, goblet cells were absent. In a recent study, a tissue engineered conjunctival equivalent was developed from cultured AM epithelial cells [45]. The conjunctival equivalent contained PAS-positive cells, indicative of goblet cells, and successfully restored the conjunctiva in a rabbit model. Transplants containing goblet cells could also be developed from nasal mucosa, which harbors goblet cells [46]. Thus, there are multiple possible cell sources for developing conjunctival equivalents, though no comparative studies have defined the optimal choice of donor cells.

A number of different substrates and membranes have been attempted for tissue engineering conjunctival epithelial equivalents. These can be categorized into: (1) biological membranes; (2) extracellular matrix protein-containing membranes; and (3) synthetic polymer membranes.

### 4.1. Biological Membranes

Seventy-six years after it was first used in ophthalmology, AM, which constitutes the innermost layer of the fetal membranes, has a prominent role in ocular surface reconstruction [47]. AM is particularly suited for clinical use as it supports epithelialization [48], reduces scaring [49], suppresses the immune response [50], reduces pain, and decreases inflammation [51]. Prior to AM transplantation (AMT), the AM is cryopreserved, which kills all the AM cells [52]. Hence, AM grafts function primarily as a matrix and not by virtue of transplanted functional cells. The membranes have most commonly been cryopreserved in a basal cell medium at −80 °C [53], but a technique for freeze-drying the AM has also been developed [54]. Freeze-dried AM can be sterilized by gamma-irradiation [54], however, AM treated this way may release a less amount of growth factors than conventionally cryopreserved membranes [55]. In addition, the AM can be sterilized with per-acetic acid/ethanol and air-dried [56]. The latter technique is, on the other hand, reported to yield inferior results compared to cryopreserved AM with respect to rate of cell outgrowth, release of wound-healing factors, and preservation of the AM basement membrane (BM) [57]. In patients with chronic inflammation there is a tendency for recurrent shrinkage and symblepharon formation after restoring the ocular surface with AM [58]. The success of transplanting AM is therefore dependent on the underlying disease [4].

Twelve studies have described culture of CEC on AM, of which eight used denuded AM (dAM) (Table 1). Meller *et al.* first reported the use of dAM for cell culture of CEC since they noticed that the devitalized AM epithelium inhibited adhesion and growth of the CEC [59]. All later studies using intact AM have utilized explant culture.

Eight out of ten studies confirmed the presence of goblet cells on AM (detected either by their mucin content or by Ck7), irrespective of whether dAM or intact AM had been used [59]. Data on actual percentages of goblet cells in CEC cultures on AM are sparse, although one study reported that between 25% and 75% of the cells were MUC5AC positive [60]. Although Ck7 positive goblet cells have been demonstrated under serum-free conditions, addition of 10% FBS improved the preservation of goblet cells [61]. This is in line with a study showing that FBS promotes expression of conjunctival epithelial cytokeratins due to the effect of vitamin A [62]. Development of mucin-containing goblet cells have also been achieved on AM independent of feeder cells, air-lifting or high calcium [60]. Thus, AM generally promotes goblet cell development.

Stratified CEC were obtained in all studies using AM, except one [63]. Culture techniques to induce stratification include the use of explants, air-lifting, feeder layer, and high calcium. Air-lifting promotes cell polarity by increasing the number of microvilli, tight junctions, and hemidesmosomes in CEC cultures [59]. The molecular mechanisms involved in air-lifting include the p38 mitogen-activated protein kinase and Wnt signaling pathways [64]. Stratification was achieved when including a feeder layer [50], air-lifting [59] and/or high calcium [65] in cell cultures. 

Stratified CEC cultures were also generated on cadaveric acellular dermis (AlloDerm) coated with collagen type 4 (COL4) [65]. The latter study employed a serum-free culture protocol without feeder cells. Goblet cells, however, were not reported. Hence, except for the latter study on acellular dermis, culture of CEC on biological membranes generally promotes stratified cultures with goblet cells.

**Table 1:** Conjunctival Epithelial cells cultured on biological membranes.

Substrate (s)	Cell Species	Explant/Suspension Culture	Culture Time (Days)	Feeder Cells	Air-Lifting	High Calcium	Basal Medium	Serum	Conjunctival Donor Site	Goblet Cells	Comment	Authors
AM	Human	Explant	21	–	–	–	CNT50	FBS/AS	–	Yes (with both serum type)		Rivas *et al.* 2014
AM	Human	Explant	14	No	No	No	DMEM:F12	5% FBS	Fornix/bulbus	Yes (<25% to 75% MUC5AC+)	Stratified culture	Eidet *et al.* 2014
AM	Human	Explant	12	No	Yes	No	DMEM:F12	5% FBS	Bulbus	Yes (MUC5AC+, fever than in native conjunctiva)	Stratified culture	Tan *et al.* 2014
AM	Rabbit	Explant	8–15	3T3/No	Yes	–	–	–	Fornix	No MUC5AC−	Stratified culture (Ck3+/Ck12−)	Cho *et al.* 2014
dAM	Human	Explant	9–11	3T3	Yes	–	DMEM:F12	10% FBS	–	Yes (PAS+, increased by γSI)	Stratified culture	Tian *et al.* 2014
dAM	Human	Explant	–	No	No	–	DMEM:F12	FBS	Fornix	Yes (PAS+)	Stratified culture	Silber *et al.* 2014
dAM	Human	Suspension	5	No	No	–	KM (serum free or DMEM:F12)	0%–20% FBS	Palpebra	Yes (100% Ck7+; best preserved by 10% FBS)		Martinez-Osorio *et al.* 2009
dAM	Human	Suspension	21	3T3	Yes	Yes	KM (serum free or DMEM:F12)	FBS	–	No (MUC5AC−)	Stratified culture	Tanioka *et al.* 2006
dAM	Human	Explant	14	No	–	Yes	DMEM:F12	FBS/HS	Bulbus	–	Stratified culture	Ang *et al.* 2005
dAM	Human	Explant	12–22	No	Yes/No	Yes/No	KGM or DMEM/F12	0 or 10% FBS	Bulbus	Yes (MUC5AC detected by PCR in all groups)	Stratified culture	Ang *et al.* 2004
dAM	Human	Explant	11–15	No	No	–	KGM:F12	10% FBS	Bulbus	–	Monolayer culture	Sangwan *et al.* 2003
dAM	Rabbit	Suspension	<28	RCF	Yes/No	No	DMEM:F12	5% FBS	–	Yes (scattered MUC5AC+ cells with/without AL and RCF)	Stratified culture (increased in AL)	Meller *et al.* 1999
AlloDerm coated with COL4	Human	Suspension	18	No	Yes	Yes	MCDB 153	No	–	–	Stratified culture	Yoshizawa *et al.* 2004

AL = air-lifting; AM = human amniotic membrane; (–) = not reported; AS = autologous serum; DMEM = Dulbecco’s Modified Eagle’s Medium; 3T3 = 3T3 feeder cells; γSI = γ-secretase inhibitor; dAM = denuded AM; KM = keratinocyte medium; HS = human serum; KGM = keratinocyte growth medium; RCF = rabbit conjunctival fibroblasts; COL4 = collagen type 4; MUC5AC = mucin 5AC; Ck7 = cytokeratin 7.

### 4.2. Extracellular Matrix Protein-Containing Membranes

The conjunctival BM is a thin connective tissue membrane, which is composed of collagen type IV (collagen α1 and α2 chains), laminin (α5, β2 and γ1 chains), nidogen-1 and -2 and thrombospondin-4 [29]. It is therefore reasonable to assume that a tissue engineered CEC equivalent would benefit from being surfaced by ECM proteins. Nineteen studies have described the culture of CEC on various ECM proteins (Table 2). Collagen type 1 (COL1) was most commonly used, either in the form of a coating [66], a gel [67] or as a compressed gel [68]. The latter two forms offer the mechanical strength to transfer the cultured cells to the surgical site. In addition, fibronectin (FN), laminin (LN), Matrigel, elastin-like polymer (ELP), gelatin-chitosan and poly-l-lysine (PLL) were tried [61,66,69,70,71,72,73,74,75,76,77]. 

Goblet cells were seen when CEC were grown inside a collagen gel [78], but not always when grown as a monolayer on top of the collagen gel [78]. Compared to Matrigel, CEC grown on COL1 expressed more MUC5AC RNA than Matrigel cultures [76]. Five percent PAS positive goblet cells were detected when culturing CEC on top of a COL1:COL3 mix in serum-free medium [71]. The latter study also achieved stratification. When cultured without feeder cells, air-lifting or high calcium, the CEC formed monolayer cultures on COL1 [66]. Stratified cultures were achieved with the addition of feeder cells [67], air-lifting [67], or high calcium [76].

Matrigel is composed of LN, COL4, heparan sulfate proteoglycans, entactin, transforming growth factor (TGF), and basic fibroblast growth factor (bFGF) [71]. Cultured CEC generally form aggregates on Matrigel rather than continuous cell sheets [66,76]. In one study the aggregates contained PAS positive goblet cells [66].

Use of fibronectin, either alone or in a mix with COL1, was reported in four studies [69,70,71,72]. The CEC formed monolayer cultures [70], but the presence of goblet cells were not reported. Elastin-like polymer has been used to grow Ck7 positive cells of the cell line IOBA-NHC [79]. Gelatin-chitosan yielded stratified cultures with Ck4 positive squamous cells when using explant culture [77]. Of all the ECM protein substrates, collagen gels and compressed collagen appear the most useful for conjunctival tissue engineering due to their mechanical properties and potential promotion of goblet cell formation.

**Table 2:** Conjunctival epithelial cells cultured on extracellular matrix protein-containing membranes.

Substrate (s)	Cell Species	Normal Cells/Cells Line	Explant/Suspension Culture	Culture Time (Days)	Feeder Cells	Air-Lifting	High Calcium	Basal Medium	Serum	Conjunctival Donor Site	Goblet Cells	Comm ent	Authors
BSA:COL mix	Rabbit	Normal	Suspension	–	No	No	No	PC-1 (serum free)	No	–	–		Scholz *et al.* 2002
COL	Rabbit	Normal	Suspension	6	No	Yes/No	No	PC-1 (serum free) or DMEM:F12	No	–	Yes (3% to 4% PAS + in AL group)		Yang *et al.* 2000
COL:FN mix	Human	Normal	Suspension	–	No	No	No	KGM (serum free)	No	Bulbus	–	Monolayer culture	Cook *et al.* 1998
COL:FN mix	Human	Normal	Suspension	–	No	No	No	EpiLife	No	–	–		Gordan *et al.* 2005
COL1	Bovine	Normal	Suspension	12	No	Yes/No	–	DMEM:F12	10% FBS	Bulbus	Yes (PAS + in both AL and submerged cultures)	Stratified culture (increased by AL)	Civiale *et al.* 2003
COL1 gel	Rabbit	Normal	Suspension	7–14	No	No	No	DMEM:F12	10% FBS	Bulbus	Yes (PAS + cell within gel, PAS− on the gel surface)	Stratified culture within gel, monolayer on the gel surface	Niiya *et al.* 1997
COL1 gel with/without 3T3	Rabbit	Normal	Suspension	6	3T3/No	Yes/No	No	DMEM:F12	10% FBS	–	No (PAS−, MUC5AC−)	Stratified culture (increased by AL and 3T3)	Chen *et al.* 1994
COL1 gel with/without 3T3 or HCF	Human	Normal	Suspension	14	3T3/HCF/no	Yes	No	DMEM:F12	5% FBS	Bulbus	Yes (only with HCF)	Stratified culture (with feeder cells)	Tsai *et al.* 1994
COL1 or Matrigel	Human	ConjEp-1/p53DD/cdk4R/TERT cell line	Suspension	(3–7 days in high Ca)	3T3/no	No	Yes	KM (serum free) or DMEM:F12	10% FBS	Bulbus	Yes (MUC5AC RNA highest with COL1)	Stratified culture (COL1), aggregates (Matrigel)	Gipson *et al.* 2003
COL1, COL1: COL3 mix, LN, FN or Matrigel	Rabbit	Normal	Suspension	<14	No	No	No	PC-1 (serum free)	0 or 1% FBS	All conjunctiva	Yes (5% PAS + in serum free cultures on COL1:COL3 mix)	Stratified culture (COL1:COL3 mix)	Saha *et al.* 1996
COL1, Matrigel or COL1:Matrigel mix	Rabbit	Normal	Suspension	–	No	No	No	DMEM:F12	5% FBS	All conjunctiva	Yes (PAS + cell in cultures on COL1 and in globules on Matrigel)	Monolayer culture (COL1), aggregates (Matrigel)	Tsai *et al.* 1988
COL1:FN mix	Rabbit	Normal	Suspension	–	No	No	–	PC-1 (serum free)	No	–	–		Basu *et al.* 1998
COL4	Rat	Normal	Suspension	10	–	Yes	–	KM (serum free) or DMEM:F12	No	Palpebra	–		Yu *et al.* 2012
Compressed COL	Human	Normal	Suspension	14	No	No	No	DMEM:F12	10% FBS	–	–	Stratified culture	Drechsler *et al.* 2015
Elastin-like polymer	Human	IOBA-NHC cell line	Suspension	5	No	No	–	DMEM:F12	–	–	Yes (Ck7+)	–	Martinez-Osorio *et al.* 2009
Gelatin-chitosan	Rabbit	Normal	Explant	14	No	No	–	DMEM:F12	10% FBS	–	–	Stratified culture (Ck4+)	Zhu *et al.* 2006
LN-1, LN-β2 or COL1 gel with BCF	Bovine	Normal	Explant	14	BCF/no	No	No	KBM (serum free) or DMEM (serum)	0 or 10% FBS	Bulbus	–	Stratified culture (DMEM/10% FCS and cultures on COL1 with BCF)	Kurpakus *et al.* 1999
LN-1, LN-β2 or poly-I-Iysine	Bovine	Normal	Suspension	–	No	No	No	KBM (serum free)	No	–	–		Lin *et al.* 1999
LN-10	Human	HC0597 cell line	Suspension	–	No	No	No	KBM (serum free)	No	–	–		Lin *et al.* 2002

AL = air-lifting; BSA = bovine serum albumin; COL = collagen; (–) = not reported; DMEM = Dulbecco’s Modified Eagle’s Medium; PAS = periodic acid-Schiff; FN = fibronectin; KGM = keratinocyte growth medium; FBS = fetal bovine serum; 3T3 = 3T3 feeder cells; HCF = human conjunctival fibroblasts; KM = keratinocyte medium; LN = laminin; KDM = keratinocyte basal medium; FCS = fetal calf serum; BCF = bovine conjunctival fibroblasts.

### 4.3. Synthetic Polymer Membranes

Included in this group are polymers of glycolic acid, lactic acid, ε-caprolactone, 1,3-trimethylene carbonate, ethyl acrylate, hydroxyethyl acrylate, and methacrylic acid. One of the benefits of using these polymers is that several of them, including poly(l-lactide-*co*-glycolide) (PLGA) and poly(ε-caprolactone) (PCL), are already approved by the Food and Drug Administration (FDA) for the use in the human body for specific applications. In addition, the biodegradability of these polymers can be adjusted by controlling the ratio and choice of polymers. For instance, PLGA degrades faster than PCL. Furthermore, in contrast to biological membranes, synthetic membranes can be manufactured under sterile conditions, thereby considerably reducing the risk of transferring infectious agents to the patient. Although biodegradable polymers have been investigated at length with various types of cells, only four studies reported biocompatibility with cultured CEC [80,81,82,83] (Table 3). Three of these explored growth of CEC on polymer substrates [80,81,82], whereas one investigated the toxicity of polymer extract on cells cultured on plastic [83]. One of the studies confirmed the presence of MUC5AC positive goblet cells of comparable density to that seen when culturing CEC on AM [80]. The remaining studies did not report presence of goblet cells. The extract study showed lowest to highest viability with 50:50 poly(dl-lactide-*co*-glycolide) (PDLGA); 85:15 PDLGA and Inion GTR^TM^, respectively [83]. In cell growth studies, substrates with all three polymers demonstrated high viability [82]. Equally high viability was also seen when growing CEC on poly(ethyl acrylate-*co-*hydroxyethyl acrylate) (P(EA-*co*-HEA)) copolymers or 90:10 poly(ethyl acrylate-*co*-methacrylic acid) (P(EA-*co*-MAAc)) copolymers [81]. Interestingly, the latter two polymer substrates showed increased adhesion, proliferation and viability when hydrophobicity was increased. In contrast, Ang, *et al.* demonstrated increased proliferation when decreasing hydrophobicity of their PCL membranes [80]. The latter authors also obtained stratified cultures, which became more stratified by increasing surface hydrophilicity with NaOH. Thus, surface modification of synthetic polymer membranes can affect adhesion, proliferation, viability and stratification. Obvious advantages of synthetic polymer membranes include existing FDA approval for specific uses in the human body, high mechanical strength and biodegradability.

**Table 3:** Conjunctival Epithelial cells cultured synthetic polymer membranes.

Substrate (s)	Cell Species	Normal Cells/Cells Line	Culture Time (Days)	Explant/Suspension Culture	Feeder Cells	Air-Lifting	High Calcium	Culture Medium	Serum	Conjunctival Donor Site	Goblet Cells	Comment	Authors
50:50 PDLGA, 85:15 PDLGA or Inion GTR^TM^	Human	IOBA-NHC cell line	–	Suspension	No	No	–	DMEM:F12	10% FBS	–	–	Extract studies showing lowest to highest viability with 50:50 PDLGA; 85:15 PDLGA; Inion GTR^TM^	Huhtala, *et al.* 2008
50:50 PDLGA, 85:15 PDLGA or Inion GTR^TM^	Human	IOBA-NHC cell line	3	Suspension	No	No	–	DMEM:F12	10% FBS	–	–	High viability with all types of polymer	Huhtala, *et al.* 2007
P(EA-co-HEA) or 90:10 P(EA-co-MAAc) copolymers	Human	IOBA-NHC cell line	–	Suspension	No	–	–	DMEM:F12	10% FBS	–	–	All polymers were non-toxic, hydrophobicity increased adhesion, proliferation and viability	Campillo-Fernandez, *et al.* 2007
Ultrathin PCL	Rabbit	Normal	–	Explant/suspension	No	No	Yes	KGM (serum free)	No	–	Yes (MUC5AC+ comparable to AM)	Stratified culture (increased by NaOH); NaOH surface modification increased hydrophilicity and proliferation	Ang, *et al.* 2006
Temperature-responsive polymer, poly(N-isopropyl-acrylamide; PIPAAm)	Rabbit	Normal	10	Suspension	No	No	No	DMEM:F12	10% FBS	Fornix/ palpebra	Yes (21.5% MUC5AC+, PAS+)	Stratified culture (4–5 cell layers); proliferation rate of 38.4%; high viability; Ck4 mRNA increased with time	Yao, *et al.* 2015

AL = air-lifting; PDLGA = poly(dl-lactide-*co*-glycolide); Inion GTR^TM^ = a blend of 85:15 poly(l-lactide-*co*-glycolide) (PLGA) and 70:30 poly(l-lactide-*co*-1,3-trimethylene carbonate) (PLTMC) copolymers in a major ratio of 70:30; DMEM = Dulbecco’s Modified Eagle’s Medium; FBS = fetal bovine serum; P(EA-*co*-HEA) = poly(ethyl acrylate-*co*-hydroxyethyl acrylate); P(EA-*co*-MAAc) = poly(ethyl acrylate-*co*-methacrylic acid); PCL = poly(ε-caprolactone); (–) = not reported; MUC5AC = mucin 5AC; PAS = periodic acid-Schiff; Ck4 = cytokeratin 4.

## 5. Future Avenues for Developing Tissue Engineered Conjunctival Epithelial Equivalents

### 5.1. Comparative Studies of the Effect of Different Substrates on Cultured Conjunctival Epithelial Cells

In 2010, Rama and associates described the importance of the phenotype for clinical success following transplantation of cultured limbal epithelial cells [84]. p63, which is a marker for undifferentiated cells, was a significant predictor of clinical outcome [84]. It is possible that the phenotype of cultured CEC will determine success following transplantation of CEC. Comparative studies on how various substrates affect the cell sheet with regard to the phenotype in particular are, therefore, warranted.

### 5.2. Storage and Transportation of Cultured Conjunctival Epithelial Cells

With steadily stricter regulations for cell therapy, which lead to centralization of culture units [85], storage technology of cultured CEC has become increasingly important to allow the tissue to be transported to eye clinics worldwide [86]. Keeping in mind the significance of the phenotype for clinical outcome [84], assessment of the phenotype among other parameters prior to surgery should ideally be performed during the storage period. Moreover, storage in a hermetically sealed container enables microbiological assessment [87]. Finally, storage technology has the advantage of offering increased flexibility in scheduling surgery [88]. Comparative studies on how various substrates influence the ability to store cultured CEC with regard to morphology, viability, and phenotype should be performed to enable worldwide access to cultured CEC.

## 6. Conclusion

Amniotic membrane is the most commonly used substrate for CEC culture. The majority of the studies demonstrated that AM support the growth of goblet cells, in contrast to several alternative substrates. A major weakness in the current literature is the lack of comparative studies, thus such studies should be prioritized to be able to identify the most ideal substrate for ocular surface repair. Considering the disadvantages inherent to the use of a foreign biological material such as AM, clinical studies involving alternative membranes should be carried out as currently only AM has so far been used for transplanting tissue engineered CEC in humans.
